# Supplementary material for: Comparative Transcriptome Analysis Revealed the Key Genes Regulating Ascorbic Acid Synthesis in Actinidia
Source: Int J Mol Sci. 2021 Nov 29;22(23):12894. doi: 10.3390/ijms222312894 (PMC8657573; doi:10.3390/ijms222312894)
Supplement: Supplementary file 1 [file ijms-22-12894-s001.zip › ijms-1447782-supplementary/Supplemental Figure.pdf]

Supplemental Figure

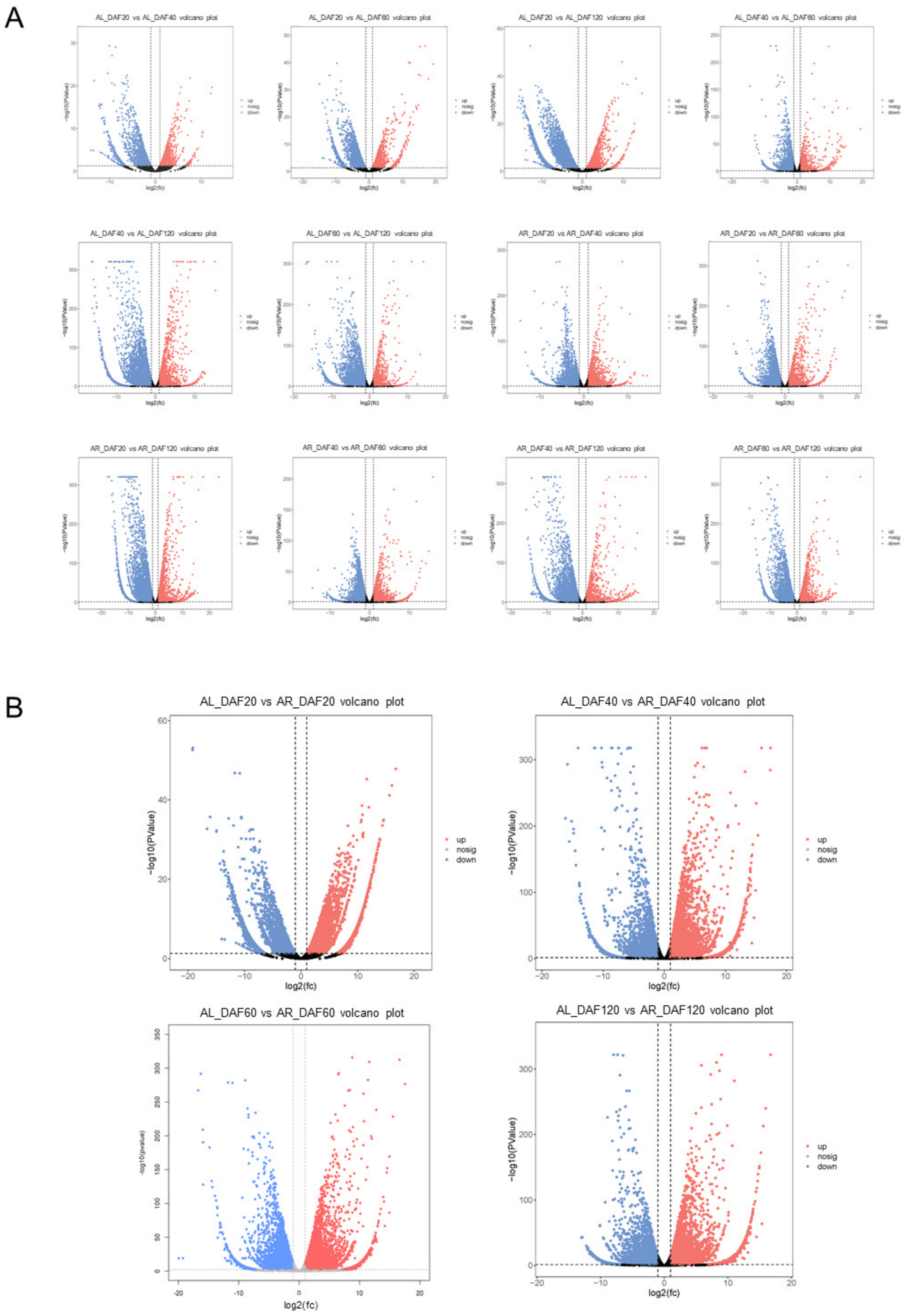

Figure S1 Volcano plots depicting differentially expressed genes. (A) Volcano plots of DEGs of the same kiwifruit species at different developmental stages. (B) Volcano plots of DEGs in different kiwifruit species at the same developmental stage. In each plot, the X-axis shows the log base 2-fold change, and the Y-axis indicates the adjusted p values for differences in expression. Red dots, blue dots and grey dots equate to upregulated, downregulated and nonsignificant, respectively.

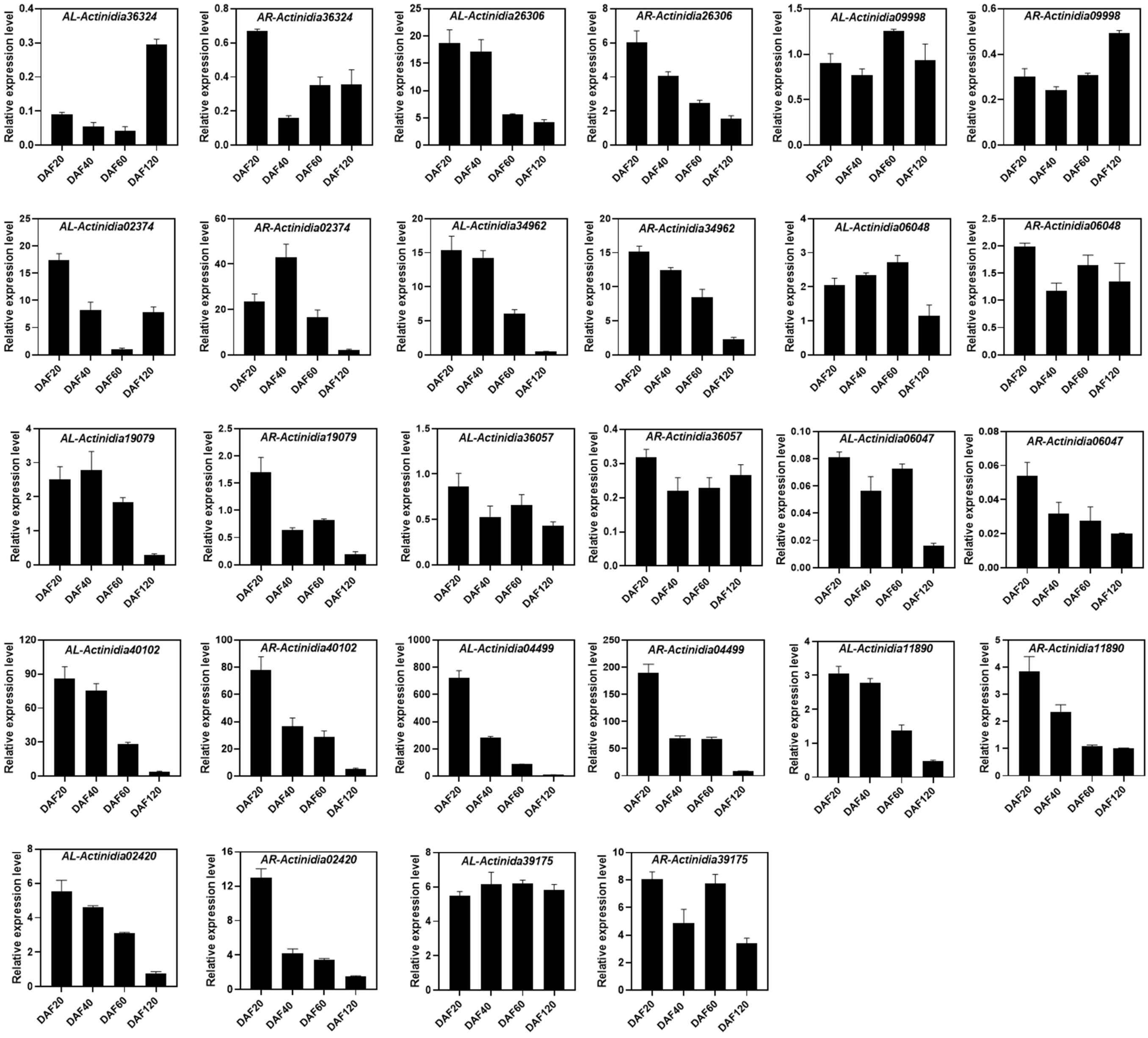

Figure S2 The expression level of genes of L-galactose pathway determinate by Real-time quantitative PCR during different developmental stages of *A. latifolia* and *A. rufa*.

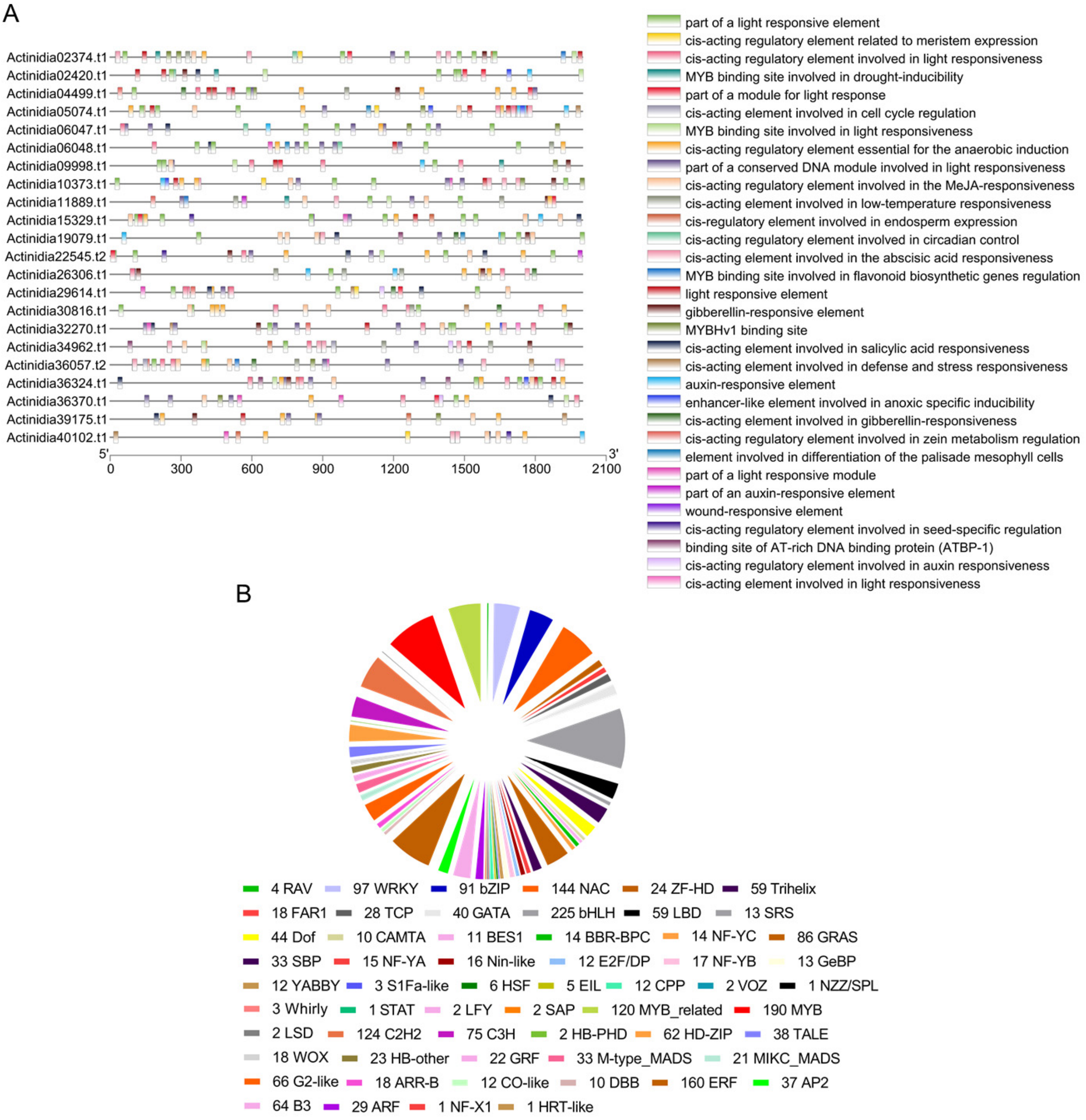

Figure S3. Prediction of cis-acting elements (**A**) of related gene promoters and related transcription factors (**B**) in the L-galactose pathway.

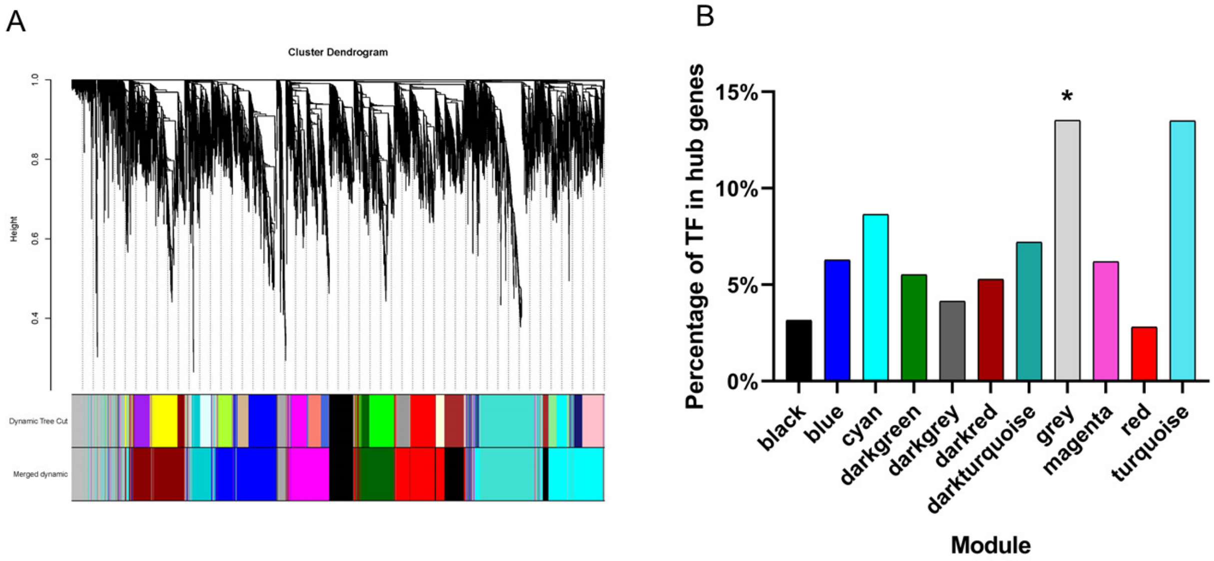

Figure S4 Coexpression network during kiwifruit development. (**A**) Weighted gene coexpression network analysis (WGCNA) analysis of RNA sequencing (RNA-seq) data from different stages of kiwifruit development. (**B**) The abundance of transcription factors of hub genes in different modules was analysed.

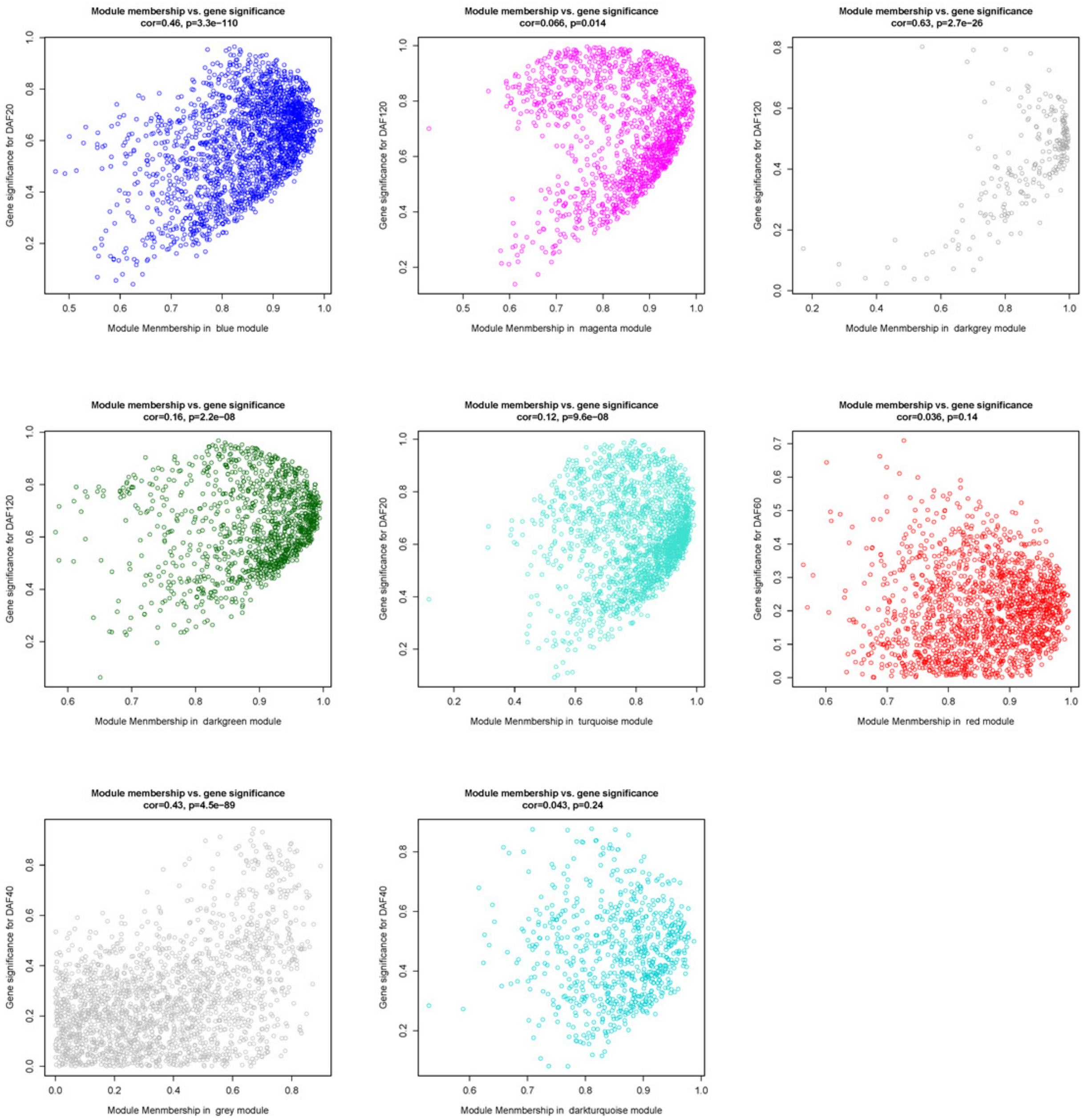

Figure S5 The significance of genes in each module at different developmental stages and the correlations of genes in each module were analysed.

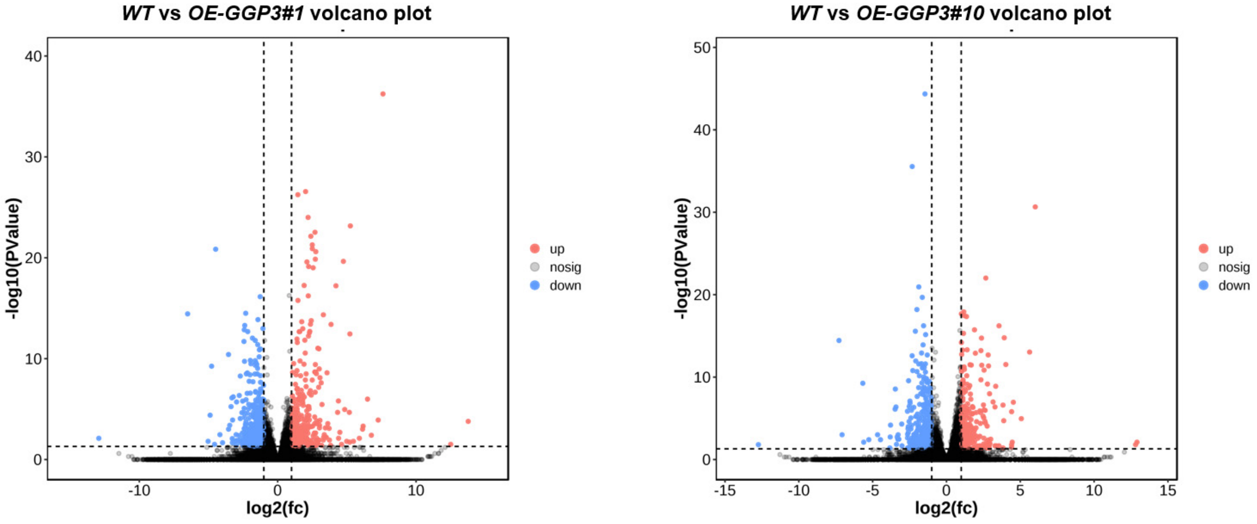

Figure S6 Volcano plots depicting differentially expressed genes between wild-type (WT) and AcGGP3-overexpressing transgenic lines.
